# Parapneumonic Empyema Deaths during Past Century, Utah

**DOI:** 10.3201/eid1501.080618

**Published:** 2009-01

**Authors:** Jeffrey M. Bender, Krow Ampofo, Xiaoming Sheng, Andrew T. Pavia, Lisa Cannon-Albright, Carrie L. Byington

**Affiliations:** University of Utah School of Medicine, Salt Lake City, Utah, USA

**Keywords:** complicated pneumonia, Spanish flu, Streptococcus pneumoniae, methicillin resistant Staphylococcus aureus, Utah Population Database, vaccine, historical review

## Abstract

Bacterial pneumonia with empyema is a serious complication of influenza and commonly resulted in death during the 1918 influenza pandemic. We hypothesize that deaths caused by parapneumonic empyema are increasing in Utah once again despite advances in critical care and the availability of antimicrobial drugs and new vaccines. In this study, we analyzed the historical relationship between deaths caused by empyema and influenza pandemics by using 100 years of data from Utah. Deaths caused by empyema have indeed increased from 2000–2004 when compared with the historic low death rates of 1950–1975. Vaccine strategies and antimicrobial drug stockpiling to control empyema will be important as we prepare for the next influenza pandemic.

## Abstract 2

Vaccine strategies and antimicrobial drug stockpiling to control empyema will increase preparedness as we prepare for the next influenza pandemic.

An influenza pandemic is thought to be the most likely and most severe biological emergency facing the United States (*1*–*3*). Bacterial pneumonia is a serious complication of influenza infection and was likely a major cause of the excess deaths seen during the 1918 influenza pandemic (*4*–*8*). Even today, though the overall disease mortality rate due to infectious diseases is declining in the United States, death from pneumonia and influenza remains one of the top 10 causes of death overall (*9*,*10*).

Parapneumonic empyema, a serious complication of bacterial pneumonia frequently caused by *Streptococcus pneumonia* and *Staphylococcus aureus*, is increasing in North and South America, Europe, and Asia (*11*–*21*). As we prepare for an influenza pandemic, changes in the microbiology of pneumonia and the increasing rates of empyema must be considered. In this study, we analyzed the historical relationship between deaths due to empyema and influenza pandemics using 100 years of data from Utah.

## Methods

### Utah Population Database

The Utah Population Database (UPDB) is a computerized genealogical database linking multiple data sources. The resource includes genealogical records of the original Utah pioneers (members of the Church of Jesus Christ of Latter Day Saints) who settled in Utah in 1847 and their descendants. These records have been linked to disease data for Utah, including death certificate records dating back to 1904. We used a version of the database without individual identifiers that spans 100 years (1904–2004).

### Identification of Patients

The institutional review boards for both the University of Utah and the UPDB reviewed and approved this study. Each death record in the UPDB contains a primary cause of death that was coded with the International Classification of Diseases (ICD) nomenclature. All death certificates between 1956 and 2004 were encoded by using ICD revisions 6 through 10. For death certificates from 1904 through 1955, a nosologist with a University of Utah research project used the literal information and coded cause of death to ICD revision 10 using the 2000 Medical Data System and supplemented this system with hand coding. During 1985–1995, the Bureau of Vital Statistics added selected secondary causes of death, including pneumonia, empyema, and influenza. Beginning in 1996, death certificate records contained multiple secondary causes of death and classified persons with each. We searched the UPDB for deaths associated with empyema on the basis of ICD codes: ICD-10 J869, ICD-9 510.9, ICD-8 510, ICD-7 518, and ICD-6 518. For influenza deaths, we used the following ICD codes: ICD-10 J10, J11, ICD-9 487, ICD-8 470–474, ICD-7 480–483, and ICD-6 480–483.

### Statistical Analysis

We analyzed empyema-related deaths by decade and population to examine trends in death related to empyema. We analyzed empyema deaths in 2 age groups: children (0–18 years of age) and adults (>18 years of age). Utah population was determined based on national census data. Utah’s population data have been available every 10 years from 1900 through 2000 and for 2005. Using available data, we fitted a cubic curve of log-transformed population for 1 year to estimate Utah’s population for other years. We defined 3 notable periods in Utah history. Period 1, 1917–1920, represents the Spanish influenza pandemic. Period 2, 1950–1975, represents the post–antimicrobial drug era and encompasses smaller influenza pandemic periods of 1957–58 and 1968–69. Period 3, 2000–2004, includes the period of increasing incidence of empyema in children (*15*,*22*) and recent increases in empyema deaths. A Poisson model was used to estimate risk for death in different years and compare estimates among the 3 identified periods.

## Results

Empyema and influenza death rates in Utah over a 100-year period are shown in the Figure. A high rate of deaths caused by empyema occurred during 1900–1909. Deaths caused by empyema peaked at 18/10,000 person-years during the decade 1910–1919, which included the 1918–19 Spanish influenza pandemic (period 1). Empyema deaths steadily decreased in the decades after the Spanish influenza pandemic. This decline is most apparent in the 1930s, coincident with the widespread introduction of sulfonamide antibiotics. With the introduction of penicillin during World War II, deaths caused by empyema leveled off substantially in the 1940s–1950s. During the period from 1950 through 1975 (period 2), empyema deaths remained at a nadir of 0.4–0.8/10,000 person-years. The rate of deaths increased significantly during the final 5-year period from 2000–2004 (period 3). Compared with period 2, the death rate for persons with empyema in Utah during period 3 was 3.2/10,000 person years, >6-fold higher (rate ratio 6.6; 95% confidence interval 3.2–13.4: p<0.005).

**Figure:**
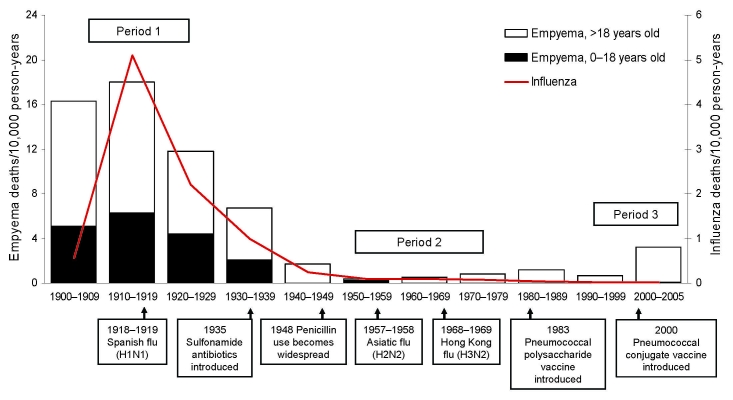
Average rates of deaths in Utah caused by parapneumonic empyema and influenza, by decade, 1900–2005.

During the pre–antimicrobial drug era and during the 1918–19 influenza pandemic, children 0–18 years of age accounted for 31%–37% of all empyema deaths. Between 1940 and 1999, deaths among children decreased more dramatically than among adults and accounted for <4% of all empyema deaths. Almost all (97%) of the empyema deaths seen recently in period 3 are in adults >18 years of age, most among persons >65 years of age.

Death rates attributed to influenza peaked dramatically during the decade 1910–1919, associated with the peak in empyema deaths. After 1970, the number of influenza deaths declined steadily and has remained very low.

## Discussion

The rates of empyema leading to death in Utah have significantly increased between 2000–2004. This increase occurred in the absence of a major influenza pandemic and in spite of advances in medical care. The recent increase in empyema deaths is unexplained and may have broad implications for future influenza pandemics.

Empyema caused substantial illness and death in the era before antibiotics. In the 8th edition of his book, published just before the Spanish influenza pandemic, Osler described empyema as “a most common complication [of pneumonia] occurring in 2.2 percent of clinical cases” seen over an 8-year period at Johns Hopkins Hospital (*22*). He described pneumococcus as the most common pathogen leading to empyema. Pneumonia and empyema were, at the time, “the most fatal of all acute diseases” (*22*). Osler himself died of pneumonia and empyema in 1919.

Influenza has historically been linked to pneumonia deaths. According to our study, deaths caused by empyema peaked during the Spanish influenza pandemic of 1918–19. The estimated worldwide death toll attributed to Spanish influenza has been estimated at 21–50 million (*23*). Pneumonia, especially due to *S. pneumoniae*, is thought to be a major contributor to the excess deaths seen during the 1918 influenza pandemic (*5*,*6*). As seen in the Figure, data from the UPDB demonstrate an increase in deaths caused by empyema during this period, although it is not as dramatic as the increase in deaths attributed to influenza. This finding suggests that empyema contributed to the deaths from pneumonia associated with the influenza pandemic.

The number of deaths due to empyema fell moderately during the decades of the 1920s and 1930s but fell dramatically after 1940 and World War II. This decrease in empyema deaths corresponds with the beginning of the antimicrobial drug era. During the 1950s and 1960s, when the pandemics of 1957 and 1968 occurred, the rate of deaths caused by empyema remained low. We did not observe a significant increase in the deaths caused by influenza over the same period. This lack of an increase may have been due to the relatively mild nature of these pandemics compared with the 1918 pandemic, the availability of antimicrobial drugs, improvements in the management of pneumonia and its complications, and perhaps the insensitivity inherent in examining death rates by decade.

We noted a statistically significant increase in the number of empyema deaths in Utah at the turn of the 21st century (2000–2004) when compared with the mid-20th century (1950–1975). This increase in empyema deaths has taken place without the advent of an influenza pandemic and in the setting of continued advances in medical care. The increase in death rates/person years caused by empyema is primarily seen in the adult population and is most apparent in patients >65 years of age. What might account for this increase in empyema deaths? We speculate that increased mortality rates from empyema are caused by changes in the organisms that cause pneumonia and empyema worldwide.

*S. pneumoniae* is thought to have been the major cause of death from secondary bacteria in prior pandemic influenza outbreaks. Comparing all 3 of the influenza pandemics of the twentieth century, 1 study estimates that 50% of bacterial pneumonia cases associated with influenza were caused by *S. pneumoniae* (*24*). Experimental as well as epidemiologic data support the association between serious *S. pneumoniae* infections and influenza. Studies by McCullers and others have shown that influenza virus infection preferentially predisposes mice to fatal infections with *S. pneumoniae* (*25*–*27*). Recent studies have demonstrated a clear temporal association between seasonal influenza and invasive pneumococcal disease in children (*28*).

Changes in circulating serotypes of *S. pneumoniae* have been reported from many regions worldwide (*11*,*14*,*16*,*17*,*29*–*32*). These changes may play a role in the increase in deaths caused by empyema. The annual incidence of invasive disease caused by *S. pneumoniae* has decreased significantly in all age groups with the introduction of the 7-valent pneumococcal conjugate vaccine (PCV-7) in 2000 (*33*). Further, disease caused by resistant *S. pneumoniae* has also decreased significantly with the introduction of the PCV-7 vaccine, which targeted the resistant serotypes (*33*,*34*). Thus, antimicrobial drug resistance to *S. pneumoniae* seems unlikely to be responsible for the increase in empyema deaths since the introduction of PCV-7. In spite of the recent decreases in invasive pneumococcal disease, hospitalizations for empyema are increasing in US children (*20*). Recent reports further show that the incidence of empyema due to non–PCV-7 serotypes, especially types 1, 3, and 19A, has increased significantly worldwide in the post PCV-7 era (*11*–*17*,*21*,*32*). These serotypes are historically associated with severe invasive disease, particularly empyema, and might contribute to the increased rates of deaths caused by empyema among adults (*17*,*35*,*36*).

*S. aureus* is increasingly recognized as a significant cause of complicated pneumonia. A recent study from France demonstrated a mortality rate of >50% in patients infected with *S. aureus* that contained Panton-Valentine leukocidin (PVL), which caused necrotizing pneumonia (*37*). A study among children from Houston demonstrated a marked increase in complicated pneumonia in patients with PVL-containing *S. aureus* infection (*29*). The PVL gene is now considered one of the identifying features of community-associated methicillin-resistant *S. aureus* (CA-MRSA) and is at least a marker for invasiveness and virulence, although the exact contribution of PVL remains unclear (*38*).

CA-MRSA has become more widespread over the past decade and has been responsible for increasing amounts of severe complicated pneumonias in the community (*19*). The Centers for Disease Control and Prevention reported a significant increase in CA-MRSA pneumonia associated with influenza infection in the United States during 2006–2007, including deaths among previously healthy children (*18*).

History reminds us that we must be prepared to deal with severe bacterial pneumonia when planning for future influenza threats. Good evidence exists that influenza will interact with bacterial pathogens to cause severe pneumonia and increased mortality rates. Thus, the recent increase in deaths caused by empyema has potential implications for pandemic influenza preparedness. The rise of pneumococcal serotypes with a propensity to cause complicated pneumonia and increasing rates of community acquired pneumonias due to CA-MRSA should be considered when developing strategies to prevent and treat influenza complications. These strategies might include broadening recommendations for existing or enhanced pneumococcal vaccines that cover serotypes associated with empyema, such as 1, 3, and 19A. Determining who should be vaccinated with these pneumococcal vaccines during an influenza pandemic would have to be done on the basis of risk and the availability of vaccines. Further, the stockpiling of antimicrobial drugs active against CA-MRSA and other resistant pathogens may also be needed. Currently, the US Department of Health and Human Services Pandemic Influenza Plan from 2005 does not specifically account for secondary bacterial infections or the need for bacterial vaccines (*1*,*39*).

However, the Infectious Diseases Society of America called for improved antibacterial agents and vaccines as a key need in pandemic influenza preparedness (*40*). Our data provide support for this concept. Extending the range of conjugate pneumococcal vaccines to include the serotypes now commonly associated with empyema and encouraging broader use of the polysaccharide vaccine may help lessen the effects of *S. pneumoniae* infection on a pandemic. If high vaccination rates cannot be routinely achieved, stockpiles of pneumococcal vaccine might be needed. Antimicrobial drug stockpiling, particularly agents with activity against MRSA, should be included in the discussion of antiviral drug stockpiling as well. Further, because the treatment for empyema frequently requires drainage either through chest tubes or surgical procedures, planning for these healthcare resources should also be considered.

This study has some limitations. Cause of death in death certificate data is not always accurate, and the accuracy may vary by disease. The clinical manifestations of empyema were well described by 1900 (*22*). Empyema was common during the pre–antimicrobial drug era and relatively easy to document with clinical examination and chest radiographs. Thus, it seems unlikely that recognition or reporting of empyema changed with time during the period covered in this historical study. There is no reason to suspect that empyema was more readily diagnosed in the final 5 years of the study, that definitions changed, or that it was more readily listed as a cause of death. Influenza, in contrast, is more difficult to diagnose clinically and may have been more readily listed as a cause of death during known pandemics and after virologic testing became available. Our data do not include microbiology results, so the association between empyema deaths and specific organisms is speculative.

We observed a significant increase in deaths caused by bacterial empyema during the period of the influenza pandemic of 1918–19 and an unexplained increase from 2000 through 2005. As secondary bacterial pneumonia historically has been a significant cause of illness and death in influenza pandemics, understanding the recent increase in empyema deaths is critical as we prepare for the next influenza pandemic. Changes in prevalent bacteria, including *S. pneumoniae* serotypes and the virulence of *S. aureus,* should be further explored. Pneumococcal vaccines targeting the serotypes most associated with empyema and antimicrobial agents against resistant bacteria such as CA-MRSA should be key components in national and international influenza pandemic planning.
